# The antipsychotic potential of *Salix Mucronata* on ketamine-induced rats

**DOI:** 10.1016/j.ibneur.2024.06.003

**Published:** 2024-06-14

**Authors:** Ntombifuthi P. Ngubane, Musa V. Mabandla, Brenda Z. De Gama

**Affiliations:** a Discipline of Clinical Anatomy School of Laboratory Medicine and Medical Sciences College of Health Sciences University of KwaZulu-Natal Westville Campus, Private Bag X54001, Durban 4000, South Africa; b Discipline of Physiology School of Laboratory Medicine and Medical Sciences College of Health Sciences University of KwaZulu-Natal Westville Campus, Private Bag X54001, Durban 4000, South Africa

**Keywords:** Herbal medicine, Mental disorders, Schizophrenia, Risperidone, *Salix mucronata*

## Abstract

*Salix mucronata* is one of the herbal plants offered by the traditional health practitioners in KwaZulu-Natal, South Africa for the treatment of schizophrenia. This study aimed to investigate the effects of repeated administration of ketamine on social interaction, novelty and motivation in adult, male Sprague Dawley rats. It also aimed to investigate the potential of risperidone and the herbal extract of *S. mucronata* to reverse impairments that are induced by ketamine. Experimental rats (n=45) received a dose of ketamine at 30 mg/kg via intraperitoneal injection for 5 consecutive days. They were then allocated into their respective treatment groups and given risperidone (APD) and the herbal extract of *S. mucronata* (TM) at doses of 6 mg/kg and 5 mg/kg, respectively, for 7 consecutive days. Social behaviour was tested using the 3-chambered sociability test, and anhedonia was tested using the sucrose preference test. Ketamine induction elicited social withdrawal and reduced social novelty which were later successfully reversed by risperidone and *S. mucronata*. The rats showed reduced preference to sucrose post-induction and post-treatment. Ketamine and mild stress caused by scruff restraint elicited reduced weight gain for the animals. No differences were noted on brain mass between controls and experimental groups and also between risperidone and *S. mucronata* groups. However, reduced brain volume was noted in experimental groups. Dopamine and acetylcholine concentration levels were high in groups which received risperidone and *S. mucronata*. These findings highlight that the antipsychotic potential of *S. mucronata* is similar to risperidone.

## Introduction

Approximately 1 in every 8 individuals are affected by mental disorders globally, with approximately 1 in 300 or almost 25 million individuals affected by schizophrenia (World Health Organisation, 2022, de Bartolomeis et al., 2023). Schizophrenia is classified as one of the most serious mental disorders due to its complicated behavioural and cognitive symptoms (Gao et al., 2023) and the reduced life expectancy of individuals diagnosed with this disorder, which is 10–25 years less compared to the general population (Sheybani-Arani et al., 2023). The symptoms of this disorder include hallucinations and delusions (positive symptoms), asociality, anhedonia, blunted affect and alogia (negative symptoms) and impairment of working memory, attention and executive function (cognitive symptoms) (Granger et al., 2023). According to Ngubane and De Gama (2023), the symptoms of schizophrenia may overlap with the symptoms observed in patients reported to be bewitched or who have an ancestral calling. This is in accordance with the study by van der Zeijst et al. (2021) who reported the aforementioned symptoms of schizophrenia as symptoms also observed in individuals with an ancestral calling.

Western treatment options for schizophrenia include medication, psychosocial rehabilitation, family interventions and psychoeducation (WHO, 2022). The medication includes antipsychotic drugs which are commonly classified into typical (first generation) and atypical (second generation). Typical antipsychotic drugs attenuate the positive symptoms whilst lacking the ability to improve the negative and cognitive symptoms of schizophrenia (Luther et al., 2023, Shettima et al., 2023). Atypical antipsychotic drugs exhibit the efficacy in alleviating both the positive and negative symptoms of this disorder (Sheybani-Arani et al., 2023). Although atypical antipsychotic drugs are reported to result in less severe side effects compared to the typical drugs, all antipsychotic drugs are associated with adverse side effects ranging from a dry mouth or mild sedation (minor side effects), to akathisia, constipation and sexual dysfunction (unpleasant effects), to acute dystonias (painful effects), to tardive dyskinesia or weight gain (disfiguring effects) and to agranulocytosis or myocarditis which are categorised as life-threatening side effects (Osacka et al., 2022, Sheybani-Arani et al., 2023). Hence, the sustained efforts in exploring alternative interventions particularly for the negative symptoms since they affect approximately 40 % of patients diagnosed with schizophrenia and prove to be the most challenging (Velligan and Rao, 2023).

Various models for the pathophysiology of schizophrenia exist; however, the dysregulation of dopamine (DA) is regarded as the primary neurotransmitter landmark and the nigrostriatal, mesolimbic, mesocortical and tuberoinfundibular pathways are the four major dopaminergic pathways (Sonnenschein et al., 2020, de Bartolomeis et al., 2023). Dopamine plays a primary role in reward processing and a dysregulation in the reward circuit results in anhedonia (Lambert et al., 2018, Sonnenschein et al., 2020). In the mesolimbic pathway, excess production of DA from the ventral tegmental area (VTA) to the limbic areas results in the development of the positive symptoms of schizophrenia (Patel et al., 2014). On the other hand, the negative symptoms of schizophrenia are associated with low levels of DA from the VTA to the cortex in the mesocortical pathway (Patel et al., 2014, Shettima et al., 2023, Velligan and Rao, 2023). The blockage of D2 receptor in the mesocortical, nigrostriatal, and tuberoinfundibular pathways has also been reported to aggravate the negative symptoms of schizophrenia (de Bartolomeis et al., 2023). Risperidone (RIS), one of the four atypical antipsychotic drugs, is established to successfully treat negative symptoms of schizophrenia through its antagonist role on D2 DA and 5-hydroxytryptamine (5-HT) 2 A receptors (Nguyen et al., 2023, Ramos-Méndez et al., 2023). However, the side effects of this drug include elevated prolactin levels, akathisia and extrapyramidal symptoms (Lang et al., 2023). Hence, the exploration of alternative medicine is warranted. The dysfunction in cholinergic systems is also linked with the pathophysiology of schizophrenia as acetylcholine plays a significant role in functions such as memory and learning processes (which are impaired in this disorder) handled in the hippocampus (de Bartolomeis et al., 2023).

In a recent study by Ngubane and De Gama (2023), the findings suggested that the commonly used approach for treating mental illness, particularly schizophrenia was through the administration of an herbal concoction. In a subsequent study, which aimed to investigate the plants (their phytochemicals and cytotoxicity) used by the THPs in herbal concoctions, THPs at two muthi (traditional medicine) markets (Berea and Dalton) recommended seven medicinal plants used or prescribed for the treatment of schizophrenia. These herbal plants included: *Dioscorea dregeana* (Kunth) T. Durand (tuber), *Boophone disticha* (bulb), *Afroaster hispida* (*Aster bakerianus*) (roots), *Salix mucronata* (Thunb) (bark), *Artabotrys monteiroae Oliv.* (roots), *Trimeria grandifolia* (Hochst) Warb. subsp. (roots) and *Clivia miniata* (Lindl.) (roots). *D. dregeana* has been reported to treat insanity, hysteria, epilepsy and psychosis (Maroyi, 2022). *B. disticha* was reported to treat unspecified mental disorders; however, it is also known to induce hallucinations (Bonokwane et al., 2022). A. hispida was documented in the treatment of chronic headaches and psychiatric disturbances (Kose et al., 2015, Mugomeri et al., 2016, Kose, 2017). *A. monteiroae Oliv.* and *T. grandifolia* were reported to treat hysteria (Mhlongo and Van Wyk, 2019) and unspecified mental disorders (Masondo et al., 2019), respectively. *S. mucronata* has been reported to treat headaches by El-Sawi et al. (2020); however, this herbal plant has not been previously reported to treat schizophrenia. Hence, the aim of this study was to investigate and document the effects of *S. mucronata* and risperidone on impaired social interaction and anhedonia in a ketamine-induced rat model.

## Materials and methods

### Animals

This study was divided into three phases; viz. adaptation (pre-induction), induction and treatment phases. A total of 66 male adult Sprague-Dawley rats (weighing between 200 and 300 g), at the beginning of the ketamine injection regime) were obtained from the Biomedical Research Unit of the University of KwaZulu-Natal. The rats were divided into two main groups, a control (Ctrl) group which received sterile saline (n=21) and an experimental (Exp) group which received ketamine (n=45) during the induction phase. From these groups, the rats were further subdivided into six groups for the treatment phase; viz. Ctrl^SAL^, Ctrl^APD^, Ctrl™, Exp^SAL^, Exp^APD^ and Exp™ (where; SAL = saline, APD = antipsychotic drug and TM = traditional medicine). The animals were single-housed in order to avoid contact with each other (Sanavi et al., 2022). However, the cages were placed close to each other to maintain auditory and olfactory contact between the animals, thus minimising stress and aggression (Becker and Grecksch, 2004). The housing conditions were set at a 12-h light/dark cycle with lights on from 6:00 AM to 6:00 PM and the room temperature was kept constant at relative humidity. All experiments were conducted during the light phase. The rats were afforded seven days of acclimatization, monitored and gently managed by the primary investigator daily. All stipulated research protocols were adhered to in accordance to the guidelines approved by the Animal Research Ethics Committee of the University of KwaZulu-Natal (Ethics Clearance number: AREC/000/5188/2023). This study also received permission from the Department of Agriculture, Land Reform and Rural Development of the Republic of South Africa (Permit number: SDAH-Epi-23021009580).

### Treatment and drug preparation

After acclimatization to laboratory conditions, behavioural tests were conducted prior to the inducement of schizophrenia symptoms with ketamine. Ketamine, at 30 mg/kg, was diluted in 0.9 % saline and administered intraperitoneally to the experimental animals for five consecutive days to induce symptoms of schizophrenia (Becker et al., 2003). The ratio of ketamine-saline solution was calculated based on the weight of each rat for the duration of the induction phase. This solution was freshly prepared daily with the dose determined according to the weight of each rat. The control groups received sub-chronic doses of 0.9 % sterile saline (0.5 ml/kg) as the experimental group via intraperitoneal injection for five days. The animals were given 24–48 hours of withdrawal after induction.

The antipsychotic drug, risperidone (Rispacor) (RIS), and one of the herbal plants (*Salix mucronata*) used by the THPs in KwaZulu Natal for the treatment of schizophrenia symptoms, were used during the treatment phase. The duration of treatment was a week (seven days) based on the findings by Potkin et al. (2003), which indicated that improvements of symptoms in patients with schizophrenia can be observed in the first week of treatment with RIS at a dose of 6 mg/day. The animal equivalent doses for risperidone and *S. mucronata* were calculated based on the weight of the animals on each day of treatment. The stock solution for RIS was prepared by dissolving 5 mg of powdered RIS in 1000 µl of 1 % DMSO, the required dose was then diluted with sterile phosphate buffed saline (PBS) as RIS was insoluble and formed instant precipitation in water and normal saline. The stock solution of *S. mucronata* was also prepared by dissolving 5 mg of the methanol extract of this plant in 1000 µl of 1 % DMSO, distilled water was then used to dilute DMSO and as a vehicle for the required dose from this solution. The solutions were freshly prepared on each day of treatment and were administered orally via oral gavage.

### Behavioural tests (BTs)

**Fig. 1:**
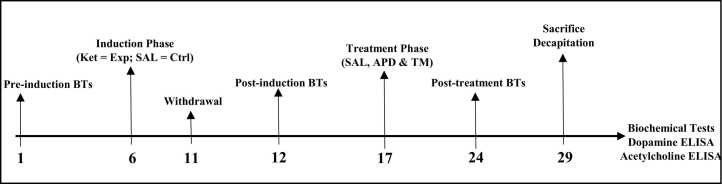
Timeline of phases of the implementation procedure.

The 3-chambered sociability and sucrose preference tests were used to test the rats for asociality and anhedonia, respectively. This was conducted pre-induction (before inducement with ketamine on the experimental group), post-induction (24–48 hours after the last ketamine injection on day five) and post-treatment with saline (SAL), Risperidone (APD) and the herbal plant methanol extract of *S. mucronata* (Fig. 1).

#### Crawley’s sociability test

**Fig. 2:**
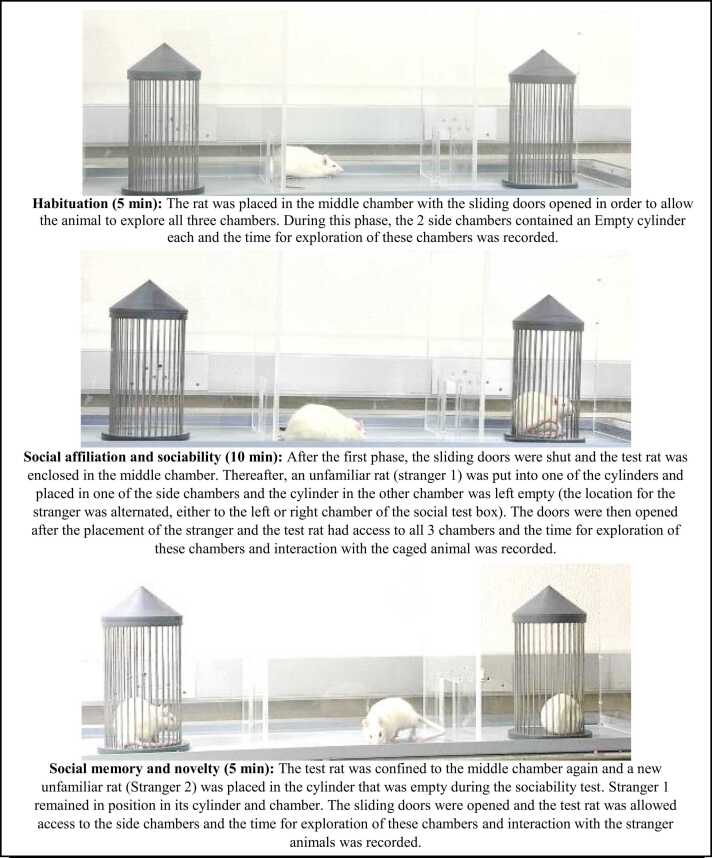
Phases of the Crawley’s sociability test.

Crawley’s sociability test also known as the three-chambered paradigm test was used to assess social interaction behaviours (Sanavi et al., 2022). The Ugo Basile sociability apparatus (with transparent walls, partitions, and lids; 120×40×40(h) cm, with 2 grid cages) was used and the protocol was adapted from Sanavi et al.’s (2022) protocol. All rats, viz. test and the 2 strangers were of the same weight, age and sex. The apparatus were cleaned using tap water and 70 % ethanol between each test. The 3 phases of this experimental procedure are depicted in Fig. 2.

During the Sociability and Novelty phase, time spent by the test animal near or interacting (sniffing, touching, biting and/or scratching) with the caged animal/s or empty cage was recorded as Time (N), and the time spent by the test animal far or not interacting with the caged animals or empty cage was recorded as Time (F). And the total time spent in each chamber with the caged animals and the empty cage was also recorded. The object Recognition Index (RI) for sociability was calculated as follows:

$$\mathrm{RI}=\frac{\mathrm{Stranger}1\;(\mathrm{TN})}{\mathrm{Stranger}1\;\left(\mathrm{TN}\right)+\mathrm{Empty Chamber}(\mathrm{TN})}$$

Where; Stranger 1 (TN) was the time spent by the test animal interacting with the caged Stranger 1 animal, and Empty Chamber (TN) was the time spent by the test animal exploring the empty cage during the sociability phase.

The RI for novelty was calculated as follows:

$$\mathrm{RI}=\frac{\mathrm{Stranger}2\;(\mathrm{TN})}{\mathrm{Stranger}2\;\left(\mathrm{TN}\right)+\mathrm{Stranger}1\;(\mathrm{TN})}$$

Where: Stranger 1 (TN) was the time spent by the test animal interacting with the caged Stranger 1 animal/familiar animal from the sociability phase, and Stranger 2 (TN) was the time spent by the test animal interacting with the novel animal/Stranger 2 introduced during the novelty phase.

The closer the RI value to 1 the more social the animals are and the more they show preference for a novelty.

#### Sucrose preference test

The two-bottle methodology for assessing sucrose preference is an effective test that can be used in the investigation of anhedonia (Eagle et al., 2016). The two-bottle experiment allows for a comparison between behavioural preferences for water to a preference for the sucrose solution. Two 100 ml bottles were placed on either side of the home cages, one with normal drinking water and one with 1 % sucrose solution. The bottles were weighed for 4 consecutive days and the side of each bottle was alternated every day to avoid side bias. The sucrose preference percentage was calculated using the formula below:

$$\%\mathrm{Sucrose Preference}=\frac{\mathrm{Consumed sucrose solution}}{\mathrm{Total liquid consumption}}\times \;100$$

### Brain morphometry

**Fig. 3:**
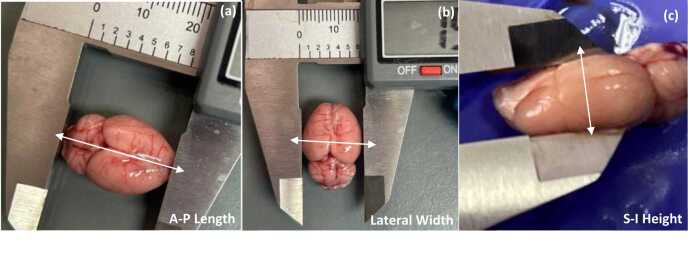
Measurements of the brain: (a) length from the most anterior part of the frontal lobe to the most posterior part of the cerebellum, (b) width from the most lateral and convex part of one side of the brain to the most lateral and convex part on the other side, and (c) height from the most superior part of the brain to the most inferior part.

After all procedures were completed, in order to avoid the interference of anaesthesia with the chemistry of the tissues, particularly neural tissue, rapid non-pharmacologic euthanasia (decapitation) was used to sacrifice the animals (Bekhbat et al., 2016). The brain was removed from the skull as per Collins et al. (2018) protocol. After removal from the skull, the brain was immediately weighed using a digital weighing balance (g) and measured using a digital Vernier caliper (mm). All measurements were conducted three times by one investigator for intra- observer reliability. A second investigator also conducted the measurements independently for inter-observer reliability. The measurements included: anterior-posterior length, lateral width and superior-inferior height (Fig. 3). All measurements were analysed for error in order to obtain the intra-observer coefficient (ICC) and Confidence Intervals (95 % CI). These measurements were then used to calculate the volume of the brain (Volume = Length × Width × Height). The frontal lobe, ventral tegmental area and the hippocampus on both the right and left sides were then dissected out from the brain for further biochemical analysis.

### Dopamine and acetylcholine ELISA

Brain tissue samples [prefrontal cortex (PFC), ventral tegmental area (VTA) and hippocampus] were weighed and rinsed and homogenised using PBS. Thereafter, the homogenates were centrifuged at 5000xg for 5 minutes and the supernatant was collected and used for further analysis. The reagents for the ELISA assays were prepared as per Elabscience® user manuals for dopamine (Catalog no. E-EL-0046) and acetylcholine (Catalog no. E-EL-0081). The kit and all samples were brought to room temperature before they were used. A total of four plates were used, two dopamine plates for PFC and VTA samples, and two acetylcholine plates for PFC and hippocampus samples.

The assay procedure for each plate was as follows: 50 μL of the standard or sample was added to each well and was followed by immediate addition of 50 μL of Biotinylated Detection Ab to each well. Thereafter, the plate was incubated at 37°C for 45 minutes. After the contents of the plate were aspirated, the plate was washed 3 times with wash buffer solution. This was followed by the addition of 100 μL of HRP Conjugate to each well and the plate was incubated at 37°C for a further 30 minutes. After the contents of the plate were aspirated again, the plate was washed 5 times using a wash buffer solution. Thereafter, 90 μL of Substrate reagent was added to each well and the plate was incubated at 37°C for 15 minutes. Lastly after incubation, 50 μL of Stop solution was added to each well and the plate was read immediately at 450 nm. The concentrations of dopamine (DA) and acetylcholine (ACh) present in each sample were determined using a standard curve.

### Data analysis

A paired samples T-test was used to analyse the findings when comparing between pre- and post-induction data or when comparing post-induction with post-treatment findings on the same group. The One-way ANOVA test was used to analyse findings between all treatment groups during all phases of the experiment. The Two-way ANOVA test was used to assess the effect and interaction between ketamine treatment and drug effect. The Post Hoc test Tukey was applied for its most attraction properties. IBM SPSS version 28.0 for Windows software and Microsoft Excel (2016) were used for data analysis and for graph presentation, respectively. Values of *p*<0.05 were considered statistically significant.

## Results

### Effect of Ketamine, Risperidone and *S. Mucronata* on Weight Gain

**Fig. 4:**
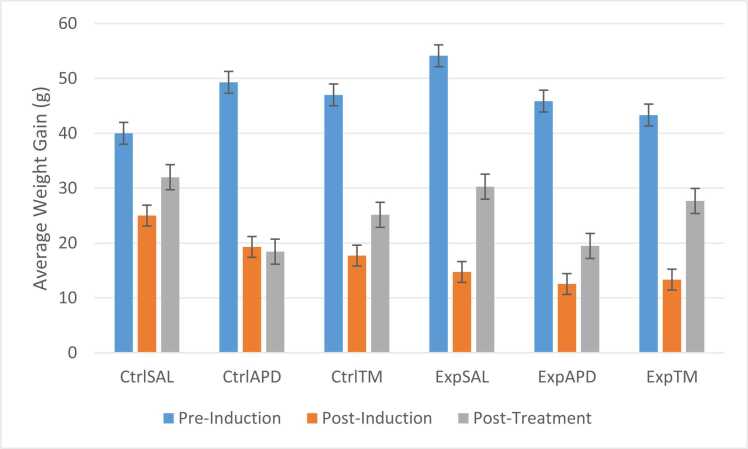
Weight gain average pre-induction, post-induction and post-treatment. The weight mean difference between the different phases (pre- and post-induction, and post-treatment were determined in order document the weight gained by animals in their groups after each phase. The weight gained by the animals was calculated as follows: Pre-induction = Adaptation Day 7 wt – adaptation Day 1 wt Post-induction = Induction Day 5 wt – induction Day 1 wt Post-treatment = Treatment Day 7 – treatment Day 1 wt No statistical significant interactions were noted for weight gained during pre- and post-induction, and post-treatment phases. The interactional effects were as follows: Pre-induction: F(2,60)=0.910, *p*=0.408; Post-induction: F(2,60)=0.202, *p*=0.818; Post-treatment: F(2,60)=0.900, *p*=0.421.

During the adaptation/pre-induction phase, the average weight gained by the rats was 40 g, 49.29 g, 47 g, 54.13 g, 45.87 g and 43.33 g for Ctrl^SAL^, Ctrl^APD^, Ctrl™, Exp^SAL^, Exp^APD^ and Exp™ groups, respectively. This dropped post-induction, the weight gain for Ctrl^SAL^, Ctrl^APD^ and Ctrl™ was 25 g, 19.29 and 17.71 g, respectively. The weight gained by the groups which received ketamine was lower compared to the control group (*p*<0.001), with the average weight gain for Exp^SAL^=14.73 g, Exp^APD^=12.53 g and Exp™=13.33 g. Post-treatment, the average weight gain increased for all groups except for Ctrl^APD^ (18.43 g), with groups Ctrl^SAL^, Ctrl™, Exp^SAL^, Exp^APD^ and Exp™ gaining 32 g, 25.14 g, 30.27 g, 19.47 g and 27.67 g, respectively (Fig. 4). The overall weight gained by the experimental groups remained lower compared to the control groups (*p*=0.003).

### Social interaction: sociability

**Fig. 5:**
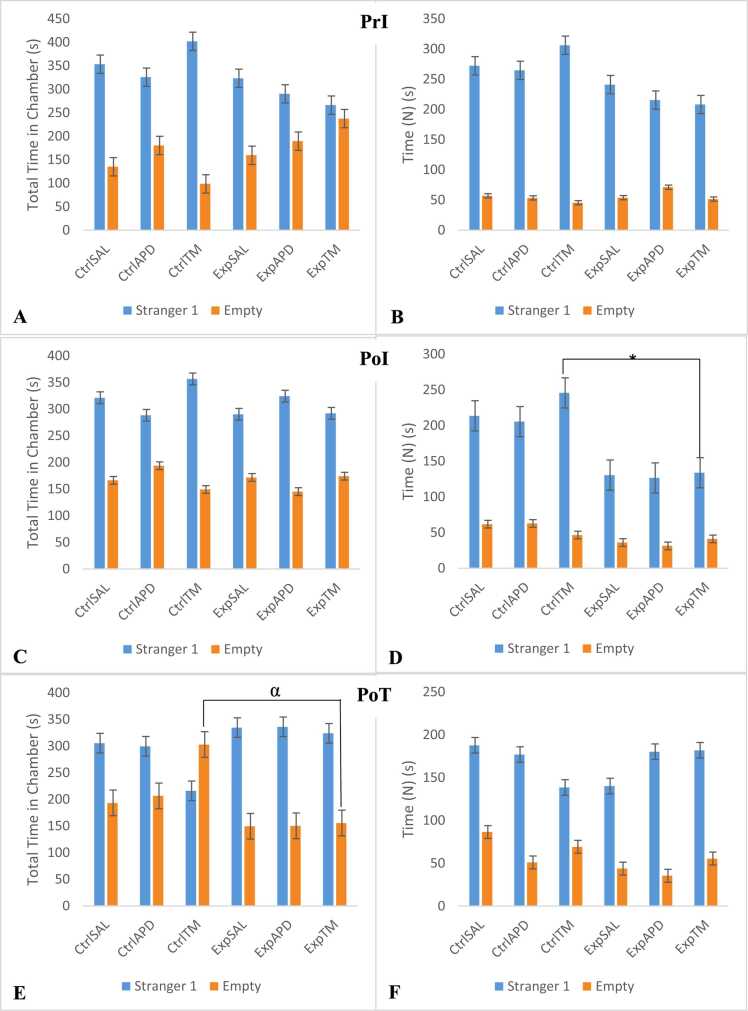
Sociability phase of the 3-chambered sociability test; (PrI, A-B) pre-induction; (PoI, C-D) post- induction and (PoT, E-F) post-treatment. After 5 min of habituation, all animals (n=66) were allowed to explore the 3 chambers of the apparatus (one at a time); and a randomly selected Stranger 1 (S1) animal (1 of n=20 of animals which served as strangers and were not part of the test animals) was introduced in a cage within one of the chambers. The order of test animals to undergo the sociability test was random and the side (left or right) where Stranger 1 was introduced was also selected randomly. The other cage on the other side was left empty (E) during this phase. Findings in this Figure are presented for time spent interacting [TN, or Time (N)] with the empty cage or cage with S1 for the pre- and post-induction, and post-treatment phases. Total time (TT) spent in each chamber with these cages was also noted. Statistical significance was noted in post-induction Time (N) in chamber with Stranger 1 for Ctrl™ vs Exp™, *p*=0.003 (*), and in post-treatment total time spent in chamber with empty cage for Ctrl™ vs Exp™, *p*=0.038 (α). No statistical significant interaction was noted for the sociability phase; however, marginal significance (**) was noted for time spent interacting with S1 post-treatment. The interactional effects for this phase were as follows: Pre-induction: S1(TN) – F(2,60)=0.535, *p*=0.588; S1(TT) – F(2,60)=0.815, *p*=0.448; E(TN) - F(2,60)=0.159, *p*=0.853; E(TT) - F(2,60)=0.979, *p*=0.382 Post-induction: S1(TN) – F(2,60)=0.400, *p*=0.672; S1(TT) – F(2,60)=1.207, *p*=0.306; E(TN) - F(2,60)=0.914, *p*=0.406; E(TT) - F(2,60)=0.815, *p*=0.447 Post-treatment: S1(TN) – F(2,60)=2.590, *p*=0.083^**^; S1(TT) – F(2,60)=0.830, *p*=0.441; E(TN) - F(2,60)=1.069, *p*=0.350; E(TT) - F(2,60)=1.368, *p*=0.263.

Based on the findings from the sociability phase of the Crawley’s sociability test, the time spent interacting with the caged animal (Stranger 1) reduced significantly post-induction for all animals which received ketamine (Fig. 5). Time (N) pre-induction for Exp^SAL^, Exp^APD^ and Exp™ groups was 241.11 s, 215.46 s and 208.09 s, respectively; which decreased post-induction to 130.51 s, 126.47 s and 133.83 s, respectively. Post-induction, statistical significance was noted between Ctrl™ vs. EXP™ for Time (N) in chamber with Stranger 1 (*p*=0.003). Statistical significance was also noted between Ctrl^APD^ vs Exp^APD^ for Time (F) in chamber with Stranger 1 post-induction (*p*=0.007). Post-treatment, Time (N) increased slightly for Exp^SAL^ to 140.13 s and increased significantly for Exp^APD^ and Exp™ to 180.24 s and 181.88, respectively. It was also noted that Time (N) for control groups also decreased post-induction; however, not as sharply as observed for the experimental groups. Time (N) pre-induction for Ctrl^SAL^, Ctrl^APD^ and Ctrl™ was 272.27 s, 264.67 and 306.28 s, respectively; post-induction this decreased to 213.55 s, 205.41 and 245.72, respectively. Post-treatment, Time (N) continued to decrease for all control groups to 187.74 s, 176.91 and 138.50 s for Ctrl^SAL^, Ctrl^APD^ and Ctrl™, respectively. Ctrl™ spent more time on chamber with an empty cage (302.89 s) compared to the total time spent on the chamber with Stranger 1 (215.95 s), and a statistical significance was noted between Ctrl™ and Exp™ (*p*=0.038) for total time spent in the chamber with an empty cage. The sociability RI decreased post-induction and decreased further post-treatment for all control groups, the decrease was particularly noticeable for Ctrl™. Pre-induction RI for Ctrl^SAL^, Ctrl^APD^ and Ctrl™ was 0.84, 0.83 and 0.87, respectively. Post-induction, RI for Ctrl^SAL^, Ctrl^APD^ and Ctrl™ was 0.77, 0.79 and 0.66, and post-treatment RI for these groups was 0.70, 0.68 and 0.58, respectively. For experimental groups, RI pre-induction for Exp^SAL^, Exp^APD^ and Exp™ was 0.80, 0.70 and 0.70, respectively. Post induction and post-treatment the RI for these experimental groups was 0.79, 0.80 and 0.74, and 0.77, 0.84 and 0.77, respectively. For Exp^APD^ and Exp™, the RI post-treatment was higher compared to RI post-induction which was higher compared to RI pre-induction. The *p*-values for sociability RI were 0.388, 0.314 and 0.042 for pre-induction, post-induction and post-treatment phases, respectively. Statistical significance was noted in the post-treatment phase. Based on the Tukey post hoc test, No statistical significance was noted between groups in the pre- and post-induction phases, statistical significance was noted between Ctrl™ vs Exp^APD^ (*p*=0.027).

### Social interaction: novelty

**Fig. 6:**
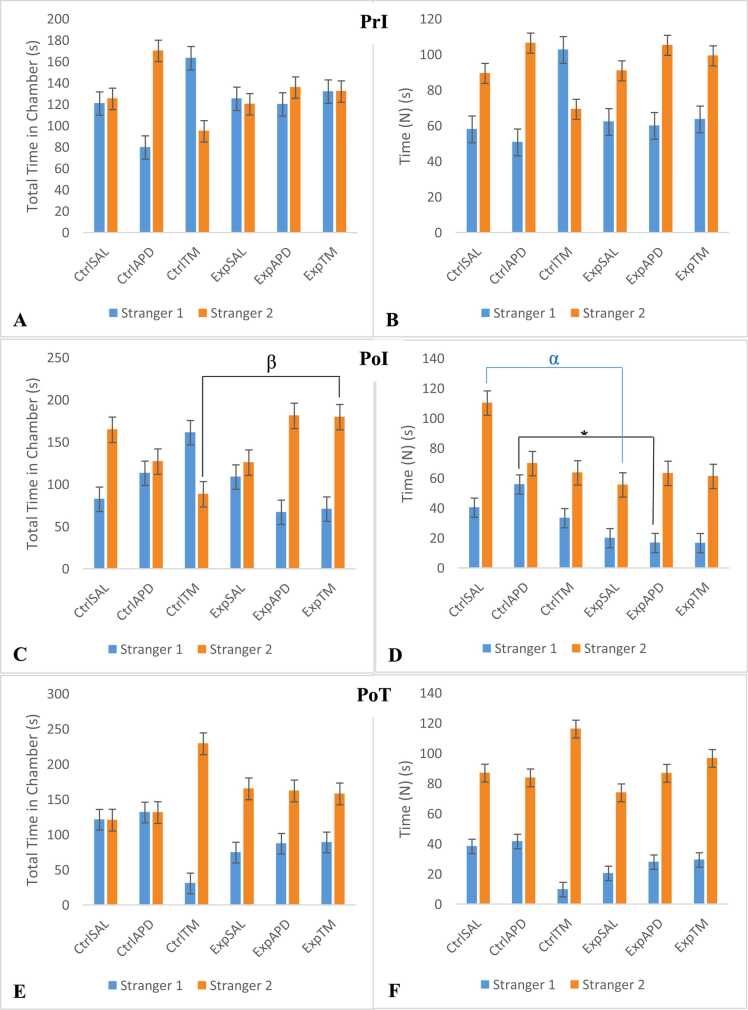
Novelty phase of the 3-chambered sociability test; (PrI, A-B) pre-induction; (PoI, C-D) post- induction and (PoT, E-F) post-treatment. After 10 min of the sociability test, all animals (n=66) were allowed to again explore the 3 chambers of the apparatus (one at a time); and a randomly selected Stranger 2 (S2) animal (also 1 of n=20 of animals which served as strangers and were not part of the test animals) was introduced in the cage which was previously empty during the sociability phase of the social interaction test. During the novelty phase (5 min), the test animal animals had access to explore or interact with either the familiar animal (Stranger 1/S1) or the novel animal (Stranger 2/S2). The test animals completed all phases of the social interaction test (habituation, sociability and novelty) before another randomly selected test animal was brought into the behavioural testing room to also complete the 3 phases. Findings in this Figure are presented for time spent interacting [TN, or Time (N)] with caged S1 and caged S2 for the pre- and post-induction, and post-treatment phases. Total time (TT) spent in each chamber with these cages was also noted. Statistical significance was noted in post-induction Time (N) in chamber with Stranger 1 for Ctrl^APD^ vs Exp^APD^, *p*=0.021 (*); post-induction Time (N) in chamber with Stranger 2 for Ctrl^SAL^ vs Exp^SAL^, *p*=0.029 (α); post-induction Total Time in chamber with Stranger 2 for Ctrl™ vs EXP™, *p*=0.047 (β). Statistical and marginal significant interactions (**) were noted post-induction and post-treatment for both time spent interacting with S1 and S2, and also total time spent in each chamber with the caged animals. The interactional effects for this phase were as follows: Pre-induction: S1(TN) – F(2,60)=0.972, *p*=0.384; S1(TT) – F(2,60)=0.820, *p*=0.445; S2(TN) - F(2,60)=0.429, *p*=0.653; S2(TT) - F(2,60)=0.942, *p*=0.396 Post-induction: S1(TN) – F(2,60)=1.017, *p*=0.368; S1(TT) – F(2,60)=3.508, *p*=0.036^**^; S2(TN) - F(2,60)=2.806, *p*=0.068^**^; S2(TT) - F(2,60)=4.750, *p*=0.012^**^ Post-treatment: S1(TN) – F(2,60)=3.715, *p*=0.030^**^; S1(TT) – F(2,60)=3.635, *p*=0.032; S2(TN) - F(2,60)=0.352, *p*=0.704; S2(TT) - F(2,60)=3.832, *p*=0.041^**^.

Based on the findings from the novelty phase of the Crawley’s sociability test, all groups preferred interacting with Stranger 2 as opposed to Stranger 1 (Fig. 6). However, the time spent interacting with Stranger 2 decreased post-induction. Time (N) pre-induction for Ctrl^SAL^, Ctrl^APD^, Ctrl™, Exp^SAL^, Exp^APD^ and Exp™ was 89.60 s, 106.62 s, 69.42 s, 91.04 s, 105.36 s and 99.48 s, respectively; post-induction this decreased to 69.98 s, 63.78 s, 55.73 s, 63.45 s and 61.39 s for Ctrl^APD^, Ctrl™, Exp^SAL^, Exp^APD^ and Exp™, respectively, while it increased to 110.41 s for Ctrl^SAL^. Post-treatment, the time spent interacting with Stranger 2 decreased to 87.10 s for Ctrl^SAL^ while it increased to 83.96 s, 116.28 s, 74.06 s, 87 s and 96.85 s for Ctrl^APD^, Ctrl™, Exp^SAL^, Exp^APD^ and Exp™, respectively. It was also noted that the time spent interacting with Stranger 1 also decreased post-induction for all groups. Time (N) for Stranger 1 pre-induction was 58.17 s, 50.86 s, 102.77, 62.34 s, 60.13 s and 63.76 s for Ctrl^SAL^, Ctrl^APD^, Ctrl™, Exp^SAL^, Exp^APD^ and Exp™, respectively. Post-induction this decreased to 40.53 s, 33.51 s, 20.16 s, 16.94 s and 16.85 s for Ctrl^SAL^, Ctrl™, Exp^SAL^, Exp^APD^ and Exp™, respectively, but for Ctrl^APD^ it slightly increased to 56.02 s. Overall, Time (N) decreased for the groups which received ketamine compared to the control groups. Post-treatment, Time (N) spent interacting with Stranger 2 for Exp^APD^ and Exp™ increased significantly to 28.08 s and 29.56 s, respectively, compared to Exp^SAL^ which showed no improvement with 20.63 s. Time (N) for Ctrl^SAL^, Ctrl^APD^ and Ctrl™ decreased to 38.51 s, 41.79 s and 9.92 s, respectively, and it decreased significantly for Ctrl™. The novelty RI increased post-induction for Ctrl^SAL^ and Ctrl™ and decreased for Ctrl^APD^. Post-treatment RI decreased for Ctrl^SAL^, it stayed more or less the same for Ctrl^APD^ and increase for Ctrl™. Pre-induction RI for Ctrl^SAL^, Ctrl^APD^ and Ctrl™ was 0.60, 0.70 and 0.37, respectively. Post-induction, RI for Ctrl^SAL^, Ctrl^APD^ and Ctrl™ was 0.76, 0.58 and 0.55, and post-treatment RI for these groups was 0.61, 0.56 and 0.94, respectively. For experimental groups, RI pre-induction for Exp^SAL^, Exp^APD^ and Exp™ was 0.59, 0.70 and 0.59, respectively. Post induction and post-treatment the RI for these experimental groups was 0.69, 0.79 and 0.79, and 0.74, 0.72 and 0.77, respectively. For Exp^SAL^ RI increased post-induction and further increased post treatment. For Exp^APD^ and Exp™, the RI increased post-induction and then decreased post treatment. The *p*-values for novelty RI were 0.424, 0.098 and 0.072 for pre-induction, post-induction and post-treatment phases, respectively. Marginal significance was noted in the post-induction and post-treatment phases. Based on the Tukey post hoc test, No statistical significance was noted between groups in the pre- and post-induction phases, marginal significance was noted between Ctrl™ vs Exp^APD^ (*p*=0.057).

### Sucrose preference

**Fig. 7:**
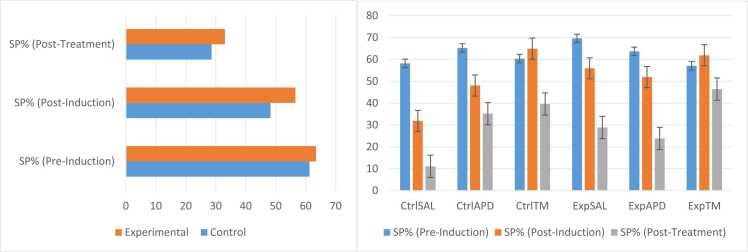
Sucrose preference percentages (SP%). Anhedonia was measured using preference for normal drinking water over a sucrose solution. All test animals (n=66) were given 2 × 50 ml bottles (one with normal drinking and water and one with 1 % sucrose solution) in their home cages for 4 consecutive days. The sides which these bottles were placed was alternated every day. Liquid consumed was measured and the sucrose preference percentages (SP%) were calculated for each day. The sucrose preference test was done for all 3 phases of the experiment (pre- and post-induction, and post-treatment). No statistical significant interactions were noted for sucrose preference. The interactional effects for this phase were as follows: Pre-induction SP%: F(2,60)=0.412, *p*=0.664; Post-induction SP%: F(2,60)=0.701, *p*=0.500; Post-treatment SP%: F(2,60)=0.877, *p*=0.421.

The preference for 1 % sucrose solution for both control and experimental groups decreased post-induction and further decreased post-treatment, with SP% for experimental groups remaining higher than that of control groups post-induction and post-treatment (Fig. 7). The SP% pre-induction for control and experimental groups was 61.26 % and 63.46 %, respectively; post-induction it decreased to 48.30 % and 56.58 %, respectively; and post-treatment it further decreased to 28.64 % and 33.05 %, respectively. Pre-induction, the SP% for Ctrl^SAL^, Ctrl^APD^, Ctrl™, Exp^SAL^, Exp^APD^ and Exp™ was 58.18 %, 65.25 %, 60.36 %, 69.60 %, 63.68 % and 57.08 %, respectively. Post-induction, the SP% for Ctrl^SAL^, Ctrl^APD^, Ctrl™, Exp^SAL^, Exp^APD^ and Exp™ was 31.89 %, 48.07 %, 64.93 %, 55.93 %, 51.90 % and 61.90 %, respectively. Post- treatment, the SP% for Ctrl^SAL^, Ctrl^APD^, Ctrl™, Exp^SAL^, Exp^APD^ and Exp™ was 11.14 %, 35.18 %, 39.61 %, 28.87 %, 23.87 % and 46.42 %, respectively. No statistical significance was noted between all groups for sucrose preference (*p*-value range between all groups was 0.230–1.000).

### Brain morphometry

**Fig. 8:**
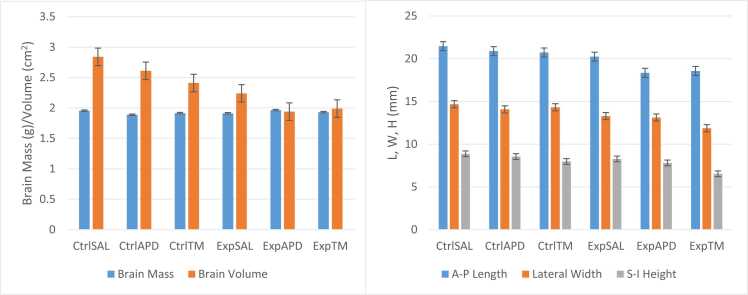
Brain morphometry. After the test animals (n=66) were sacrificed, their brains were harvested and their mass, length (L), width (W) and height (H) were measure and the volume was calculated from L, W and H. No statistical significant interactions were noted for brain morphology. The interactional effects were as follows: Mass: F(2,60)=0.167, *p*=0.846; Volume: F(2,60)=0.217, *p*=0.805; Length: F(2,60)=0.490, *p*=0.615; Width: F(2,60)=0.820, *p*=0.445; Height: F(2,60)=0.461, *p*=0.633.

The A-P length for Ctrl^SAL^, Ctrl^APD^, Ctrl™, Exp^SAL^, Exp^APD^ and Exp™ was 21.47 mm, 20.89 mm, 20.73 mm, 20.24 mm, 18.35 mm and 18.57 mm, respectively (intra-observer ICC=0.871; 95 % CI: 0.678–0.948; inter-observer ICC=0.935; 95 % CI: 0.875–0.971). The lateral width for Ctrl^SAL^, Ctrl^APD^, Ctrl™, Exp^SAL^, Exp^APD^ and Exp™ was 14.68 mm, 14.08 mm, 14.32 mm, 13.29 mm, 13.12 mm and 11.87 mm, respectively (intra-observer ICC=0.899; 95 % CI: 0.752–0.959; inter-observer ICC=0.946; 95 % CI: 0.896–0.976). The S-I height for Ctrl^SAL^, Ctrl^APD^, Ctrl™, Exp^SAL^, Exp^APD^ and Exp™ was 8.88 mm, 8.56 mm, 7.98 mm, 8.28 mm, 7.81 mm and 6.54 mm, respectively (intra-observer ICC=0.788; 95 % CI: 0.481–0.914; inter-observer ICC=0.910; 95 % CI: 0.822–0.960). No statistical significance was noted for all measurements between control and experimental groups which received the same treatment solutions (*p*-value range between all groups was 0.108–0.803). However, statistical significance was noted for Ctrl^SAL^ vs. Exp^APD^ (*p*=0.024) and Ctrl^SAL^ vs. Exp™ (*p*=0.044) A-P length. There was no significant difference noted between the controls and experimental groups on brain mass (*p*- value range between all groups was 0.995–1.000); however, the brain volume varied significantly between these groups (Fig. 8). The brain mass for Ctrl^SAL^, Ctrl^APD^, Ctrl™, Exp^SAL^, Exp^APD^ and Exp™ was 1.96 g, 1.89 g, 1.92 g, 1.91 g, 1.97 g and 1.93 g, respectively. The calculated brain volume for Ctrl^SAL^, Ctrl^APD^, Ctrl™, Exp^SAL^, Exp^APD^ and Exp™ was 2.84 cm2, 2.61 cm2, 2.41 cm2, 2.24 cm2, 1.94 cm2 and 1.99 cm2, respectively.

### Biochemical analysis

The DA concentration in the PFC was higher in both the control and experimental groups which received RIS and the *S. mucronata* extract with a statistical significant difference noted between the Exp^SAL^ and Exp™ groups (*p*<0.001). The DA concentration in the VTA was also higher in the control and experimental groups which received RIS and the *S. mucronata extract*; however, there was no statistical significance noted between these groups (*p*-value range=0.065–1.000 between all groups). The ACh concentration in the PFC was higher in the control groups which received RIS and the *S. mucronata* extract with a statistical significant difference noted between Ctrl^SAL^ and Ctrl^APD^ (*p*=0.028). There was no difference noted for ACh concentration on the PFC in the experimental groups. There was also no difference noted in the ACh concentration in the hippocampus between all treatment groups; however, this concentration was lower in the Exp™ group compared to all other groups.

**Fig. 9:**
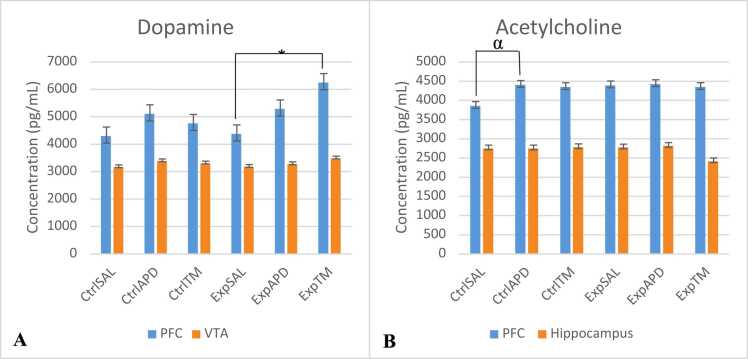
Dopamine (in PFC and VTA) and Acetylcholine (in PFC and Hippocampus) concentrations. Key: After the brain was measured for all test animals (n=66), the prefrontal cortex, ventral tegmental area and hippocampi were harvested from both sides of the brains. ELISA kits were used to determine the dopamine and acetylcholine concentrations from the harvested parts of the brain. Statistical significance was noted for DA PFC Exp^SAL^ vs Exp™, *p*<0.001 (*), and for ACh PFC Ctrl^SAL^ vs Ctrl^APD^, *p*=0.028 (α).Statistical significant interaction was noted for the PFC dopamine concentration and marginal significance was noted for PFC acetylcholine concentration. The interactional effects were as follows: DA_PFC: F(2,60)=2.606, *p*=0.082^**^; DA_VTA: F(2,60)=1.258, *p*=0.321; ACh_PFC: F(2,60)=4.181, *p*=0.020^**^; ACh_Hippo: F(2,60)=0.579, *p*=0.564.

## Discussion

The modelling of a complete symptomatic spectrum of psychiatric disorders in animals remains a challenge for researchers; hence, animal models have been extensively used to enhance understanding of this human phenomenon; by reproducing these disorders, to understand their etio-pathology and study the effects of new treatments (Geyer and Markou, 1995). The limitation in using animal models is that laboratory animals lack higher mental function as they have a simpler brain cortical structure (Bialon and Wasik, 2022). Irrespectively, selected symptoms can be modelled successfully (Ellenbroek and Cools, 2000). In this study, a pharmacological animal model for schizophrenia symptoms was created using sub-chronic administration of ketamine. Exposure to chronic ketamine has been reported to reduce appetite and weight gain in both adult rats and humans (Parise et al., 2013), which was successfully done and explained by the decrease in weight gained by the animals post-induction in the current study. The control groups also showed a decrease in weight gained post-induction even though they were injected with sterile saline and not ketamine, this could be explained by the use of scruff restraint for intraperitoneal injection which is categorised as a mild stressor that animals do not adapt to (Lindsay et al., 2021). The scruff restraint could have also impaired their locomotor response (Lindsay et al., 2021), thus reducing their food consumption leading to lower average weight gain.

The administration of ketamine led to social withdrawal effects on the experimental groups in the current study, indicating a successful model for asociality. Control groups also showed signs of social withdrawal, which were not as significant as those in the experimental groups, which might have been induced by the aforementioned mild stress caused by scruff restraint (Lindsay et al., 2021). Social isolation could be another contributing factor to the change in behaviour observed in the control groups (Shangase et al., 2023). Post-treatment, a significant reverse in social withdrawal was noted in the experiment groups which received the RIS and *S. mucronata*. Contrarily, the control groups did not show improvement in social withdrawal post-treatment. Snigdha and Neill (2008) also reported that selected antipsychotics alleviate social deficits which were induced by PCP in this study. Pre-induction, all animals showed preference for social novelty; however, the time spent interacting with Stranger 2 significantly decreased for the experimental groups post-induction. The time spent interacting with Stranger 1 also decreased post-induction. Post-treatment, a significant increase in the preference for social novelty was noted for the experimental groups which received RIS and *S. mucronata*. Furthermore, antipsychotics such as clozapine have also been reported to reverse deficits of social novelty discrimination in a study by Harich et al. (2007). Social withdrawal from Stranger 2 or social novelty improved post-treatment for the control groups which received RIS and *S. mucronata*, and the control group which received saline showed no improvement in social novelty.

Post-induction, sucrose preference for all groups decreased with the SP% for experimental groups being higher compared to control groups. This suggests that the groups which received ketamine were less anhedonic compared to the rats which did not receive ketamine. Sucrose preference continued to decrease for all groups post-treatment; however, the SP% for both the control and experimental groups which received *S. mucronata* was higher compared to other groups and the SP% for RIS receiving animals was only higher in controls. This suggests that the herbal extract had a slight potential in alleviating anhedonia which has not been reported previously.

According to Wulff et al. (2023), following chronic stress, the preference of a sucrose solution diminishes in stress-naive rodents. This might explain the continued decrease in the sucrose preference post-induction and post-treatment for all groups as all animals in the current study were exposed to some level of stress, contributed to by isolation and handling during the induction and treatment phases and also during behavioural tests. The further decrease in sucrose preference post-treatment can also be explained by findings by Gres et al. (2023) stating that antipsychotic medication can induce anhedonia by increasing the sensitivity to DA in the reward pathway. Nevertheless, a single administration of a sub-anaesthetic dose of ketamine has been reported to possess antidepressant effects in treatment-resistant depressed patients (Zanos and Gould, 2018); thus, the anhedonic-state in the experimental groups post- induction might have been alleviated by the sub-chronic administration of ketamine in the current study. Antidepressants influence anhedonia as this symptom is common in depressive disorders and is also one of the persistent negative symptoms of schizophrenia (Gres et al., 2023).

There was no difference between the brain mass across all control and experimental groups. However, a decrease in brain volume was noted in all experimental groups which received ketamine. The control and experimental groups which received saline during the treatment period had a higher brain volume compared to the groups which received RIS and *S. mucronata*. These findings are similar to the observations reported by Veijola *et al.* (2014) whereby the reduction in brain volume in schizophrenia was noted and both atypical and typical antipsychotic medications were reported to also contribute to these reductions. The whole brain volume reduction was also reported in clinical high risk patients for psychosis in a meta- analysis by Vissink et al. (2022). It was also noted that the A-P length, lateral width and S-I height measurements were also lower for experimental groups compared to control groups. The control and experimental groups which received saline during treatment had higher A-P length compared to the groups which received RIS and *S. mucronata*. The experimental group which received *S. mucronata* had the lowest lateral width, while the control and experimental groups which received *S. mucronata* had the lowest S-I height. This suggests that *S. mucronata* also contributed to reduction of the brain volume as similar to RIS.

Cognitive function is regulated by the cortical DA projections and processes, while flexible behaviour, attention and working memory, are influenced by DA in the PFC (Sesack and Carr, 2002). Dopamine functionality is determined by the origin of the dopamine neurons, and neurons originating from the VTA are associated with reward prediction error (Ott and Nieder, 2019). Through the mesocortical dopamine pathway, the midbrain dopamine neurons in the VTA are projected to the PFC (Ott and Nieder, 2019). The dopamine concentration in the PFC for control and experimental groups which received RIS and *S. mucronata* were higher compared to the groups which received saline in the current study. This suggests that RIS and *S. mucronata* increased the levels of DA in the PFC which is in accordance with the findings reported by Mork et al. (2009). The negative and cognitive symptoms of schizophrenia are associated with the dopaminergic hypofunction in the PFC and second-generation APDs have been previously reported to increase the DA levels in the PFC in order to alleviate these symptoms (Mork et al., 2009). Minor differences were noted in the DA concentration in the VTA across all control and experiment groups.

The control groups which received RIS and *S. mucronata* also had higher ACh concentrations in the PFC compared to the control groups; however, no differences in this concentration were noted in the experimental groups. This suggests that RIS and *S. mucronata* are capable of increasing the ACh release in the PFC, even though this was only noted in healthy rats which did not receive ketamine in the current study. These findings concur with reports by Ichikawa et al. (2002) who demonstrated that RIS and other atypical antipsychotic drugs; such as olanzapine, ziprasidone and clozapine increased the levels of ACh in the PFC. The findings in this study showing increased ACh levels in the PFC support the noted slight increased sucrose intake in the groups which received RIS and *S. mucronata* during post-treatment behavioural tests. ACh levels in the hippocampus were not affected by RIS in the current study; however, the *S. mucronata* showed a decrease in the ACh concentration in the hippocampus of ketamine-induced rats. These findings differed from Shirazi-Southall et al. (2002) who reported that second-generation antipsychotic drugs; such as RIS, olanzapine and clozapine, increase the levels of ACh in the rat hippocampus. However, Shirazi-Southall et al. (2002) had also reported RIS and ziprasidone to produce a moderate increase of the ACh compared to olanzapine and clozapine which produced a robust increase of this neurotransmitter.

## Conclusions, limitations and recommendations

The current study provides evidence that the administration of repeated doses of ketamine in rats induces deficits in sociability and social novelty. The current study also provides evidence of the antipsychotic potential of the herbal plant, *Salix mucronata* as animals treated with this extract reversed social and novelty deficits caused by ketamine in a similar manner as RIS. The potential effects of *S. mucronata* to alleviate the negative symptoms of schizophrenia was also confirmed by the increase of DA and ACh levels in the prefrontal cortex; however, memory deficits were not affected by either the RIS and *S. mucronata*, as no improvements were noted in ACh levels in the hippocampus. These findings support the use of *S. mucronata* as a traditional medicine approaches in the management of negative symptoms of schizophrenia.

The limitations of this study included the lack of ability to track memory for reward location with the employed sucrose preference test protocol, this is recommended for future studies as it will provide clear insight on the role of RIS and *S. mucronata* in memory. Additionally, the daily food and water intake of the rats was not tracked, thus the reasons for reduced weight gain post induction can only be hypothesized based on previous literature reports. Furthermore, the isolation of animals influenced their social behaviour. Thus, isolation interfered with the activity of both risperidone and *S. mucronata*. Therefore, future studies are recommended to use models that limit social isolation. Furthermore, it is also recommended for future research to investigate different pathways of the neurotransmitters linked to the pathophysiology of schizophrenia and also investigate how the herbal plants, and their bioactive compounds, may affect these pathways and the projection of different neurons.

## CRediT authorship contribution statement

**Musa Mabandla**: Conceptualization, Supervision. **Brenda De Gama**: Conceptualization, Supervision, Writing – review & editing. **Ntombifuthi Princess Ngubane**: Conceptualization, Data curation, Formal analysis, Funding acquisition, Investigation, Methodology, Project administration, Resources, Validation, Visualization, Writing – original draft.

## Ethical considerations

All research protocols in this study were adhered to in accordance to the guidelines approved by the Animal Research Ethics Committee of the University of KwaZulu-Natal (Ethics Clearance number: AREC/000/5188/2023). This study also received permission from the Department of Agriculture, Land Reform and Rural Development of the Republic of South Africa (Permit number: SDAH-Epi-23021009580).

## Funding

The authors extend their gratitude to the Department of Higher Education and Training for the New Generation of Academics Programme (nGAP) funding and the University of KwaZulu- Natal for the University Capacity Development Programme (UCDP) of the for the year 2023 which assisted in acquiring the Ugo Basile Sociability apparatus, ELISA kits and other necessary materials required for all conducted tests.

## Declaration of Competing Interest

The authors have no conflict of interest to disclose.
